# US county-level estimation for maternal and infant health-related behavior indicators using pregnancy risk assessment monitoring system data, 2016–2018

**DOI:** 10.1186/s12963-022-00291-6

**Published:** 2022-05-21

**Authors:** Yan Wang, Heather Tevendale, Hua Lu, Shanna Cox, Susan A. Carlson, Rui Li, Holly Shulman, Brian Morrow, Philip A. Hastings, Wanda D. Barfield

**Affiliations:** 1 Division of Population Health, National Center for Chronic Disease Prevention and Health Promotion, Centers for Disease Control and Prevention, Atlanta, GA 30341 USA; 2 Division of Reproductive Health, National Center for Chronic Disease Prevention and Health Promotion, Centers for Disease Control and Prevention, Atlanta, GA 30341 USA; 3 Health Resources and Services Administration, Rockville, MD 20857 USA; 4 Far Harbor LLC, Austin, TX 78701 USA

**Keywords:** Infant non-supine sleeping position, Maternal breastfeeding, Multilevel regression, Pregnancy risk assessment monitoring system, Small area estimation

## Abstract

**Background:**

There is a critical need for maternal and child health data at the local level (for example, county), yet most counties lack sustainable resources or capabilities to collect local-level data. In such case, model-based small area estimation (SAE) could be a feasible approach. SAE for maternal or infant health-related behaviors at small areas has never been conducted or evaluated.

**Methods:**

We applied multilevel regression with post-stratification approach to produce county-level estimates using Pregnancy Risk Assessment Monitoring System (PRAMS) data, 2016–2018 (*n* = 65,803 from 23 states) for 2 key outcomes, breastfeeding at 8 weeks and infant non-supine sleeping position.

**Results:**

Among the 1,471 counties, the median model estimate of breastfeeding at 8 weeks was 59.8% (ranged from 34.9 to 87.4%), and the median of infant non-supine sleeping position was 16.6% (ranged from 10.3 to 39.0%). Strong correlations were found between model estimates and direct estimates for both indicators at the state level. Model estimates for both indicators were close to direct estimates in magnitude for Philadelphia County, Pennsylvania.

**Conclusion:**

Our findings support this approach being potentially applied to other maternal and infant health and behavioral indicators in PRAMS to facilitate public health decision-making at the local level.

Maternal and infant health indicators include a wide range of health outcomes and health-related behaviors before, during, and shortly after pregnancy. The demand for local-level (e.g., county) data related to maternal and infant health indicators is growing among state and local health departments. Data at the local level can help improve the understanding of geographic disparities in these indicators and lead to more effective planning of program and policy strategies to promote maternal and infant health. Local-level data can also help guide local health departments on which maternal and infant health issues most need to be addressed; state estimates may not reflect local needs. However, most maternal and infant data, particularly health-related behavioral indicators, are usually only available at the national or state level. Some local health departments have developed specific surveys to obtain local-level data, such as the Philadelphia Pregnancy Risk Assessment Monitoring System (PhillyPRAMS) and the Los Angeles Mommy and Baby Project [1]; however, most counties lack sustainable resources or capabilities to collect local-level data. In such cases, model-based estimation for small areas could be a feasible approach.

A variety of model-based estimation methods have been developed for small area estimation (SAE) over the past decades [2, 3], and consequently there has been a rapid increase in the use of SAE in estimating maternal and infant health indicators in recent years [4]. Most of these studies have focused on infant mortality and some infectious diseases in developing countries; most estimation was based on Bayesian hierarchical models; and estimation techniques overall are complex [4]. Estimation for health-related behaviors, such as breastfeeding duration and infant sleeping positions, is challenging, partly because the sources of survey data for SAE are limited, and independent sources of local data used for validation are rarely available.

In this study, we focused on 2 maternal and infant health-related behaviors and used a multilevel regression with post-stratification (MRP) approach to estimate county-level prevalence from the Pregnancy Risk Assessment Monitoring System (PRAMS) [5] survey data. The multilevel regression model has been increasingly used for small area estimation because it accounts for individual factors and the hierarchical nesting of data simultaneously [6–9]. MRP was originally developed by Gelman and Little to estimate state-level voter preference using national polls [10–12]. The Centers for Disease Control and Prevention (CDC) expanded this approach to produce prevalence estimates for adult chronic conditions at 4 geographic levels (county, place [incorporated and Census designated], ZIP Code Tabulation Area, and census tract levels in the United States [www.cdc.gov/PLACES]) by using health data from the Behavioral Risk Factor Surveillance System [13–15]. The objective of this study is to estimate county-level prevalence of maternal breastfeeding and infant non-supine sleep position (a risk factor for sudden unexpected infant death) in the US and evaluate how well the MRP method performs.

## Methods

### Data source

PRAMS is an ongoing state-based surveillance system of self-reported maternal behaviors, attitudes, and experiences before, during, and shortly after pregnancy [5]. It is administered by the Division of Reproductive Health (DRH) at CDC in collaboration with state health departments. The PRAMS protocol is approved by the Institutional Review Board of the CDC and by each participating PRAMS site. From each participating site, a stratified, random sample of women with a recent live birth is selected monthly from birth certificate files. Women are surveyed 2–6 months postpartum (average = 4 months) using a standardized protocol and questionnaire. In the current study, we examined 2016–2018 PRAMS data (*n* = 65,803) from 23 states (a total of 1,471 counties) that agreed to provide their data for this research. The dataset included a geographic identifier of county (the number of respondents in 1,405 counties with non-zero respondents ranged from 1 to 2,335 with a median of 10, and 66 counties did not contain any respondents) for each respondent and individual level demographic and health variables. Annually, PRAMS data for each site are weighted for sampling design, nonresponse, and noncoverage to produce data representative of the site’s birth population for the year.

Two health-related behaviors, infant sleeping position and maternal breastfeeding duration, were assessed using 2 questions from PRAMS. Respondents were asked, “In which one position do you most often lay your baby down to sleep now?” and responses were classified into 2 groups, supine sleep position (on back) and non-supine (on side or on stomach). Respondents were also asked several questions related to maternal breastfeeding. If they were currently (at the time of survey completion) breastfeeding (or feeding pumped milk) or, for those not breastfeeding at the time of survey completion, if they ever breastfed (or pumped breast milk) for ≥ 8 weeks or ≥ 2 months, then they were coded as any breastfeeding at 8 weeks; otherwise, they were coded as not. Missing values or responses with “do not know” were excluded for each indicator (*n* = 2,680 for infant sleeping position and *n* = 2,637 for maternal breastfeeding duration). We calculated the state-level weighted direct estimates for both outcomes using SUDAAN (Research Triangle Institute, North Carolina).

We obtained county-level direct estimates from PhillyPRAMS 2018, which is not a part of PRAMS but an independent survey sponsored by the Philadelphia Department of Public Health (https://www.phila.gov/departments/department-of-public-health). It provides estimates for Philadelphia County (which includes the city of Philadelphia) for indicators similar to those in PRAMS with a sample size of 1,489. The data were acquired by DRH through a data use agreement. Given identical questions on breastfeeding and infant sleep position on the 2 surveys, we defined infant non-supine sleeping position and breastfeeding for 8 weeks for PhillyPRAMS in the same way as for PRAMS data and calculated their weighted direct estimates for Philadelphia County using SUDAAN (Research Triangle Institute, North Carolina).

### Model construction

We constructed a multilevel logistic regression model for each of the binary outcomes, Y, respectively.

1 $$P(Y_{ijk} = 1) = \log {\text{it}}^{ - 1} \left( {X_{ijk} \beta + {\text{re}}_{j \left( i \right)} + {\text{re}}_{ i} } \right)$$

where

*Y*_*ijk*_: Survey response of infant non-supine sleeping position (yes or no) or breastfeeding at 8 weeks (yes or no) from respondent *k* in county *j* and state *i*

${\mathrm{X}}_{\mathrm{ijk}}$: A matrix of respondent *k*’s demographic and socioeconomic variables.

$\beta$: A fixed but unknown parameter vector for ${X}_{\mathrm{ijk}}$

${\mathrm{re}}_{j (i)}$: Random effect of county *j* nested in state *i.*

${\mathrm{re}}_{i}$: Random effect of state *i.*

${X}_{ijk}$ was a set of respondent *k*’s covariates. It initially included variables that were associated with *Y*_*ijk*_ based on literature review, such as maternal age, maternal race, maternal marital status, maternal education level, paternal education level, and infant sex and that were evaluated based on Pickering et al.’s empirical approach [16, 17]. Only those variables that showed significant associations (the square of the parameter estimate divided by the square of its standard error greater than 4) entered the model. The final variables included in the model were maternal education (less than high school, high school, some college, college and above), maternal Hispanic ethnicity (yes or no), and maternal race (white, black, Asian, and other; other included American Indian, Hawaiian, non-white other, Alaska Natives, or mixed race) for both indicators. As the county-level random effect, ${\mathrm{re}}_{j(i)}$, was not significant (*p* > 0.05) in the model for infant non-supine sleeping position, it was excluded from the final model for this outcome. If the between-state variance (variance in null model minus variance in final model [model includes random effects only] and then divided by variance in the null model, %) was greater than 40%, then we moved on to the next step of prediction and produced the estimates. We performed modeling using the SAS 9.4 GLIMMIX procedure (SAS Institute, Cary, North Carolina). The residual subject-specific pseudo-likelihood method was used to produce the parameter estimates $\widehat{\beta }$ (with variance ${\widehat{\upsigma }}_{\widehat{\beta }}$) and empirical best linear unbiased predictors, ${\widehat{\mathrm{re}}}_{j(i)}$(with variance ${\widehat{\upsigma }}_{{\widehat{\mathrm{re}}}_{j(i)}}$) and ${\widehat{\mathrm{re}}}_{i} (\mathrm{with variance }{\widehat{\upsigma }}_{{\widehat{\mathrm{re}}}_{i}})$. The covariance structure was specified as variance components. For the 66 counties in the 23 states included in this study without any respondents in PRAMS, we also generated their ${\widehat{\mathrm{re}}}_{j(i)}$ by averaging their neighboring ${\widehat{\mathrm{re}}}_{j(i)}$ so that we could provide estimated prevalence for all the counties.

Model parameters were then applied to live birth counts from National Center for Health Statistics birth certificate files, 2016–2018. To post-stratify, we categorized the live birth counts by maternal education level, maternal Hispanic status, and maternal race for each county; thus, each county had a total of 32 (4 × 2 × 4) categories. The predicted probability (${\widehat{p}}_{\mathrm{ijm}}$) of breastfeeding at 8 weeks for the *m*^th^ category in county *j,* state *i* was calculated based on the following formula:

2 $$\hat{p}_{ijm} = \exp \left( {X_{ijm} \hat{\beta } + \widehat{{{\text{re}}}}_{j\left( i \right)} + \widehat{{{\text{re}}}}_{i} } \right)/\left(1 + \exp \left( {X_{ijm} \hat{\beta } + \widehat{{{\text{re}}}}_{j\left( i \right)} + \widehat{{{\text{re}}}}_{i} } \right)\right)$$

where *X* is a matrix of demographic variables, and ${X}_{\mathrm{ijm}}$ is the row of category *m,* and ${X}_{ijm}\widehat{\beta }={\widehat{\beta }}_{\mathrm{maternal\,education}}+{\widehat{\beta }}_{\mathrm{maternal}\,\mathrm{hispanic}}+{\widehat{\beta }}_{\mathrm{maternal}\,\mathrm{race}}$. The formula for the probability of infant non-supine sleeping position was similar to (2) but omitting ${\widehat{\mathrm{re}}}_{j(i)}$. With ${\widehat{p}}_{ijm}$, we could calculate the estimated prevalence of the indicator through post-stratification for state *i* and for county *j* as below:

3 $$\begin{gathered} \hat{P}_{i} = \sum \left( {\hat{p}_{ijm} *N_{ijm} } \right)/\sum N_{ijm} \hfill \\ \hat{P}_{j} = \sum \left( {\hat{p}_{ijm} *N_{jm} } \right)/\sum N_{jm} \hfill \\ \end{gathered}$$

where $\sum {N}_{ijm}$ is the live birth counts of state *i*, and $\sum {N}_{jm}$ is the live birth counts of county *j*. To take into account the uncertainty arising from the models, we adopted Monte Carlo simulation, a tool to generate sample statistics by using point estimates of model parameters and their asymptotic covariance matrix of these estimates [18] to simulate the distribution for the estimate. Thus formula (2) became the following:

4 $$\hat{p}_{ijm} = {\raise0.7ex\hbox{${\exp \left( {X_{ijm} \hat{\beta }^{*} + \widehat{{{\text{re}}}}_{j\left( i \right)}^{*} + \widehat{{{\text{re}}}}_{i}^{*} )} \right)}$} \!\mathord{\left/ {\vphantom {{\exp \left( {X_{ijm} \hat{\beta }^{*} + \widehat{{{\text{re}}}}_{j\left( i \right)}^{*} + \widehat{{{\text{re}}}}_{i}^{*} )} \right)} {\left( {1 + \exp \left( {X_{ijm} \hat{\beta }^{*} + \widehat{{{\text{re}}}}_{j\left( i \right)}^{*} + \widehat{{{\text{re}}}}_{i}^{*} } \right)} \right)}}}\right.\kern-\nulldelimiterspace} \!\lower0.7ex\hbox{${\left( {1 + \exp \left( {X_{ijm} \hat{\beta }^{*} + \widehat{{{\text{re}}}}_{j\left( i \right)}^{*} + \widehat{{{\text{re}}}}_{i}^{*} } \right)} \right)}$}}$$

where ${\widehat{\beta }}^{*}$ is a normal variate with mean $\widehat{\beta }$ and variance ${\widehat{\upsigma }}_{\widehat{\beta }}$, ${\widehat{\mathrm{re}}}_{j(i)}^{*}$ is a normal variate with mean ${\widehat{\mathrm{re}}}_{j(i)}$ and variance ${\widehat{\upsigma }}_{{\widehat{\mathrm{re}}}_{j(i)}}$, and ${\widehat{\mathrm{re}}}_{i}^{*}$ is a normal variate with mean ${\widehat{\mathrm{re}}}_{i}$ and variance ${\widehat{\upsigma }}_{{\widehat{\mathrm{re}}}_{i}}$. Model (4) was repeated 1000 times, and then we applied each ${\widehat{p}}_{ijm}$ to formula (3), which generated 1,000 ${\widehat{P}}_{j}$. The mean and 95% confidence interval (CI, a range between the 2.5^th^ and 97.5^th^ values) were determined for breastfeeding at 8 weeks and similarly for infant non-supine sleeping position but without ${\widehat{\mathrm{re}}}_{j(i)}^{*}$ in formula (4). In a sensitivity analysis, we fit the same model but incorporated the sampling weights from the PRAMS data. We checked the performance of the models by comparing the model estimates with weighted estimates observed from the PRAMS survey file (state-level only) and PhillyPRAMS survey (county-level). All the above analysis was conducted in SAS 9.4 (SAS Institute, Cary, North Carolina).

## Results

Table 1 shows the state- and county-level random effects for breastfeeding at 8 weeks and state-level random effect for infant non-supine sleeping position in null and final models. The final model for breastfeeding at 8 weeks explained 47% of between-state variation and 51% of between-county variation. In the model for infant non-supine sleeping position, the covariates explained 65% of the state-level variance. The results were tested further by the comparison with direct estimates at both state and county levels.

**Table 1:** Proportion of area-level variance explained by the multilevel regression model for maternal and infant behavior indicators, 23 States, 2016–2018

Random effect	Null model ^a^		Full model ^b^		% area-level variance explained
	Area-level variance	SE	Area-level variance	SE	
Breastfeeding at 8 weeks					
State	0.188	0.050	0.099	0.020	47
County	0.063	0.005	0.031	0.004	51
Infant non-supine sleeping position ^c^					
State	0.046	0.008	0.016	0.003	65

*Abbreviation* SE, standard error

^a^Model with random effects only

^b^Model with both random and fixed effects

^c^Model only included state-level random effect

In Table 2, we listed 2 sets of estimates for the indicators by state. The median model estimate of breastfeeding at 8 weeks was 67.8% and ranged from 46.8 to 82.0% among 23 states. Model estimates were very close to the direct survey estimates for each state. The Pearson correlation coefficient (Pearson’s *r*) between the 2 sets of estimates was 93%. The 95% CIs of model estimates were wider than those of the corresponding direct estimates. The median model estimate of infant non-supine sleeping position was 18.4%, ranged from 12.6 to 29.9%, and was very close to direct estimates for each state as well, but the 95% CIs were narrower than the direct estimates. The Pearson’s *r* between the 2 sets of estimates was 93%. We also observed that the 95% confidence intervals of maternal breastfeeding duration state estimates were wider than those of infant sleeping position.

**Table 2:** State- and County-level estimates (%) of maternal and infant behavior indicators, 23 States, 2016–2018

	Survey sample size	Maternal breastfeeding at 8 weeks (%)		Infant non-supine sleeping position (%)	
		Direct estimate (95% CI)	Model estimate (95% CI)	Direct estimate (95% CI)	Model estimate (95% CI)
*State *^*a*^					
AL	3233	51.3 (49.2, 53.3)	50.1 (46.2, 54.1)	27.9 (26.0, 29.9)	26.6 (24.6, 28.6)
AK	2340	80.6 (78.9, 82.3)	79.4 (76.1, 82.4)	20.6 (18.9, 22.4)	20.8 (19.1, 22.6)
CO	3881	78.2 (76.5, 79.8)	77.4 (74.5, 80.2)	12.9 (11.6, 14.2)	14.3 (13.0, 15.5)
GA	1727	59.2 (56.0, 62.4)	56.4 (52.5, 60.2)	26.2 (23.4, 29.2)	25.1 (23.1, 27.2)
IL	3912	67.1 (65.4, 68.7)	65.9 (61.9, 69.1)	18.2 (16.9, 19.6)	18.9 (17.2, 20.3)
KY	1455	56.7 (53.3, 60.0)	57.5 (53.2, 61.7)	15.3 (13.1, 17.8)	16.4 (14.8, 18.2)
LA	2634	48.0 (45.9, 50.0)	46.8 (43.2, 50.8)	32.1 (30.2, 34.0)	29.9 (28.0, 31.9)
ME	4170	72.2 (69.9, 74.3)	69.7 (65.8, 73.3)	12.3 (10.8, 14.0)	12.6 (11.3, 14.0)
MD	2227	74.6 (72.4, 76.7)	73.2 (69.4, 76.7)	22.7 (20.7, 24.8)	22.5 (20.7, 24.5)
MA	2505	71.0 (69.2, 72.8)	73.8 (70.4, 76.9)	15.8 (14.6, 17.1)	17.7 (16.3, 19.2)
MI	5565	64.3 (62.6, 65.9)	62.6 (59.2, 66.1)	17.4 (16.2, 18.7)	17.1 (15.9, 18.4)
MN	3557	76.6 (74.9, 78.2)	73.3 (70.0, 76.3)	13.6 (12.3, 15.0)	15.1 (13.8, 16.5)
MO	3105	62.5 (60.5, 64.5)	61.9 (58.2, 65.4)	18.4 (16.9, 20.1)	18.4 (16.9, 19.9)
NV	3571	66.8 (62.6, 70.7)	67.8 (61.9, 73.3)	21.1 (17.7, 24.9)	20.6 (18.4, 22.9)
NM	1327	70.6 (69.1, 72.1)	69.9 (66.1, 73.3)	20.1 (18.8, 21.5)	20.5 (18.9, 22.2)
OR	2989	83.4 (81.1, 85.4)	82.0 (79.1, 84.6)	15.1 (13.3, 17.2)	14.8 (13.5, 16.2)
PA	3240	63.3 (61.2, 65.3)	61.9 (58.2, 65.3)	17.7 (16.2, 19.4)	17.3 (15.9, 18.8)
RI	3336	65.8 (63.9, 67.6)	61.3 (56.0, 66.4)	17.9 (16.4, 19.4)	16.0 (14.4, 17.7)
SC	548	58.2 (52.5, 63.8)	57.2 (51.9, 62.5)	26.2 (21.4, 31.7)	22.8 (20.1, 25.7)
SD	1727	75.5 (73.2, 77.8)	72.0 (68.3, 75.6)	12.7 (11.1, 14.4)	12.7 (11.3, 14.2)
TN	1743	59.1 (55.9, 62.1)	55.9 (52.0, 60.0)	21.1 (18.6, 23.8)	19.9 (18.1, 21.7)
UT	4101	78.5 (76.9, 80.0)	77.3 (73.8, 80.5)	14.1 (12.8, 15.4)	14.6 (13.3, 15.9)
VA	2910	70.2 (67.5, 72.8)	71.6 (68.1, 74.8)	21.4 (19.1, 23.8)	19.0 (17.3, 20.6)
*County *^*b*^					
Philadelphia	1489	65.9 (61.6, 70.0)	58.8 (53.1, 64.1)	23.9 (20.3, 28.0)	(22.1, 25.9)

*Abbreviations*: CI, confidence interval

^a^State-level direct estimates were derived from Pregnancy Risk Assessment Monitoring System (PRAMS) data

^b^County-level direct estimates were derived from 2018 Philadelphia Pregnancy Risk Assessment Monitoring System (PhillyPRAMS) data

**Fig. 1:**
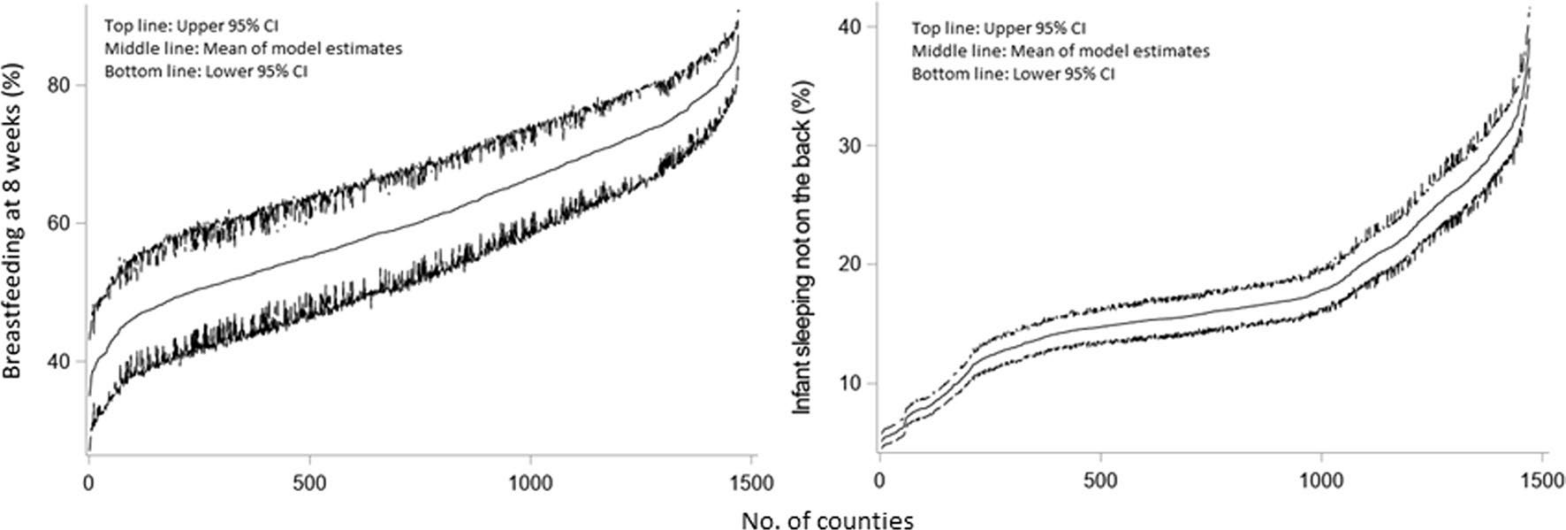
Ordered county-level model estimates (middle lines) and lower 95% confidence intervals (CIs) (bottom lines) and upper 95% CIs (top lines) for breastfeeding at 8 weeks (left plot) and infant non-supine sleeping position (right plot) among 1,471 counties in 23 states, using Pregnancy Risk Assessment Monitoring System (PRAMS) data, 2016–2018

Among the 1,471 counties from 23 states, the median model-based estimate of breastfeeding at 8 weeks was 59.8% with a range from 34.9 to 87.4%, and the median of infant non-supine sleeping position was 16.6% with a range from 10.3 to 39.0%. Figure 1 shows ordered model estimates and their 95% CIs of each outcome. The 95% CIs for maternal breastfeeding duration county estimates were found to be wider than those for infant sleeping position. In Philadelphia County (Table 2), the estimate for breastfeeding at 8 weeks was 58.8% (model, 95% CI: 53.1%, 64.1%) versus 65.9% (direct, 95% CI: 61.6%, 70.0%), and the estimate for infant non-supine sleeping position was 24.0% (model, 95% CI: 22.1%, 25.9%) versus 23.9% (direct, 95% CI: 20.3%, 28.0%). The 2 sets of estimates’ 95% CIs overlapped for both indicators.

**Fig. 2:**
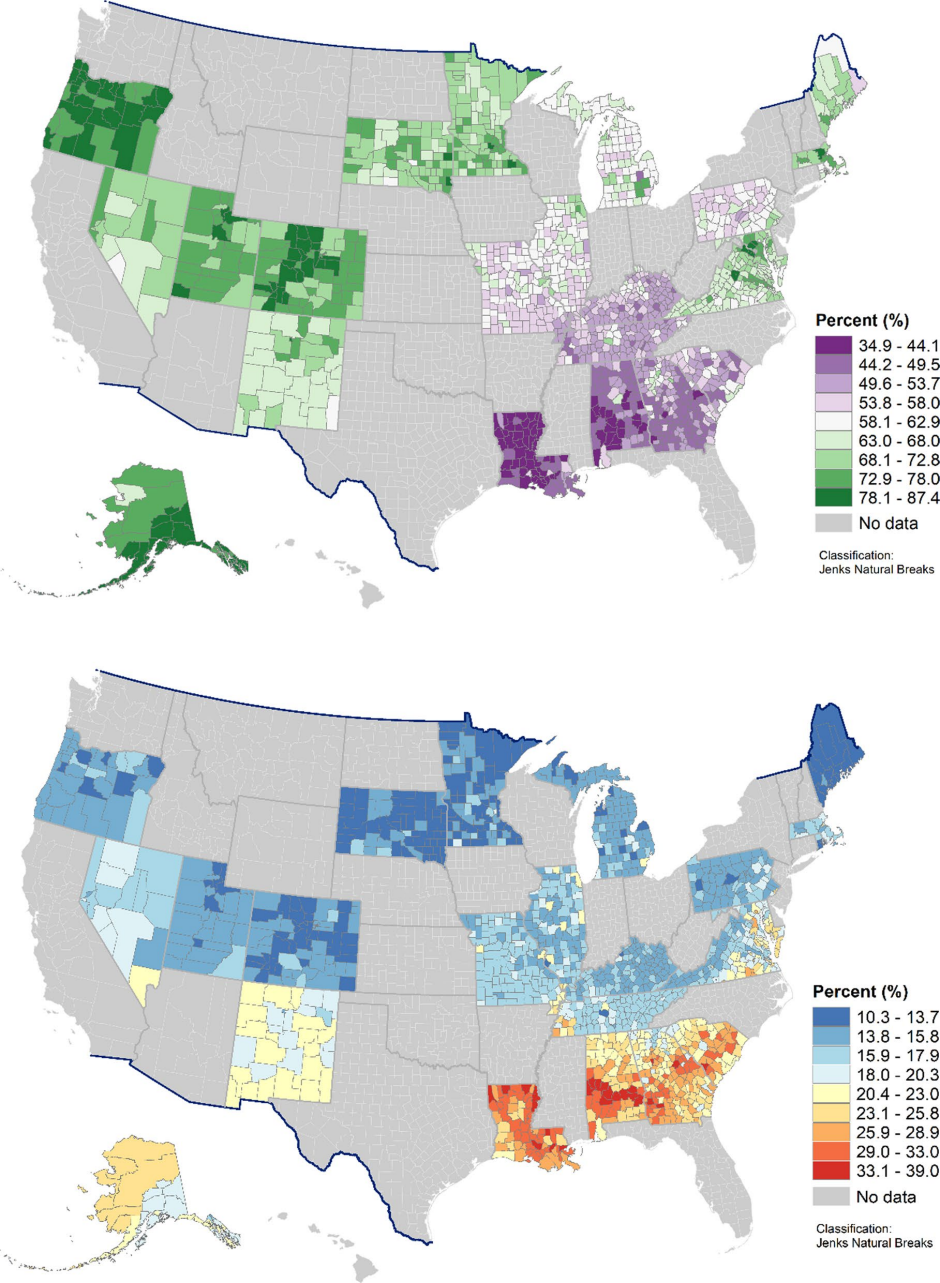
Geographic distributions of model estimates by county (1,471 from 23 states) for breastfeeding at 8 weeks (top) and infant non-supine sleeping position (bottom) using Pregnancy Risk Assessment Monitoring System (PRAMS) data, 2016–2018

Figure 2 illustrates the county-level geographic distribution of the 2 indicators among 23 states. The top map shows model estimates of breastfeeding at 8 weeks. For most of the states, the variations in prevalence among counties were small. For example, the prevalence of maternal breastfeeding at 8 weeks in Oregon was relatively high across the counties and was low across the counties in Louisiana, and the same patterns were observed in almost all the counties in the respective states. In some other states, such as Alabama, Georgia, and South Carolina, variations in the prevalence of breastfeeding at 8 weeks were seen across the counties. For example, county-level estimates varied from 38.7 to 64.2% in Alabama. Similar patterns were observed for infant non-supine sleeping position. In the sensitivity analysis, we used the sampling weights when modelling the data. Differences between the weighted and unweighted estimates were within 1% for 98% of the counties and thus were not reported.

## Discussion

In this study, we generated estimates of 2 maternal and infant health-related behaviors for counties from 23 states via an MRP approach and used direct estimates at both state and county levels to assess the validity of the model estimates.

Good performance of MRP relies on multiple factors, such as the prevalence of the indicator in the population, data sources, model specification, availability of matching populations, and quality of the data used for validation. One of the major challenges of MRP is how to specify the multilevel regression model adequately and accurately. There are no “gold standard” criteria on variable selection. Methodologists suggest including all variables that have an important impact on sampling and nonresponse and are also potentially predictive of the outcome of interest [19, 20]. However, increasing the number of variables in the model does not necessarily increase the accuracy of the model estimates, because adding variables may cause non-convergence or overfitting problems [21]. The variables considered in the model should be not only correlated with the outcome, but also able to explain much of the area-level variance [16]. The approach we adopted, which was based on Pickering et al.’s empirical experience, was straightforward and performed well according to the validation analysis.

Another challenge of MRP is assessing the uncertainty around the model estimates. Because bias and variance cannot be derived in closed form [22], the measure of uncertainty relies on resampling techniques. In Bayesian estimation a posterior distribution of small area estimates is produced, however, large datasets with complex sampling can present convergence and computational challenges [3]. Therefore, frequentist approaches, such as bootstrapping and Monte Carlo simulation [18, 23], are also used to estimate mean square error or construct 95% confidence intervals. Maternal breastfeeding estimates had somewhat wider confidence intervals than infant sleeping position estimates. Besides different outcomes and different specification of models, the extent of the between-area variation that remained unexplained in the model also played a role [24]. The confidence intervals can be used by local health departments that may wish to compare rates and estimated precision across counties, or to evaluate subgroup differences on maternal and infant health indicators [21].

External validation is typically done by comparing similarities in the distribution and correlations between model and direct estimates rather than the absolute values because multiple factors may contribute to systematic differences in absolute values. However, independent, reliable, and concurrent local surveys are rare, which makes external validation a big challenge in small area estimation. In this study, PhillyPRAMS data was available for external data validation, and absolute values and overlapping CIs were used for comparison purposes. The model estimates were quite consistent with the direct estimates for the county of Philadelphia, which is primarily due to 2 reasons. First, the variables included in the model captured important covariates for maternal and infant outcomes, which yielded good estimation results. Second, PhillyPRAMS is similar to PRAMS, including identical questions and weighting. If more county-level samples are drawn by local districts, future studies might be able to offer additional validation evidence of this methodology across a range of outcomes.

Other limitations should also be noted. First, the small area estimation process does not serve to reduce any non-sampling errors in the survey data, such as non-response or recall biases; to the extent they exist these errors remain within the modeling process. Second, although we pooled 3 survey years of data, the sample size in some counties remained small (< 10), which might pose a challenge in modelling. We are not clear how these small sample sizes influenced the predictions of the county-level random effects and subsequently the model estimates. However, a study found that although increases in sample size help improve precision of the model estimates, including relevant covariates helps even more as sample sizes become very small [25]. Further study is warranted to better understand the influence of small sample sizes related to the categorization of the random effect variable. Finally, we did not account for geographic covariates, such as health care access or hospital density, as some other studies have done when estimating maternal and infant health indictors [5]. Although there was little variation among counties after demographic and socioeconomic variables were introduced, if a strong state-level covariate was included, the model performance might be improved.

The multilevel model estimation process implemented in this study has important public health implications. While our estimation process did not explicitly include policy or program intervention effects, the analysis of county- and state-level random effects could provide insights into potential policy and practice implications. For example, infant sleeping position tended to have more variability at the state level than the county level, suggesting that policies, programs, or practices applied statewide may be sufficient to meet the needs of populations at higher levels of risk. Maternal breastfeeding may potentially be affected by a wide range of local factors, such as lactation service or support, given there was relatively more heterogeneity among counties than there was for infant sleeping position; this suggests that these data may be more helpful for determining where local interventions are most needed.

## Conclusion

Our findings support this approach being potentially applied to other maternal and infant health and behavioral indicators in PRAMS for estimates at substate levels, such as county, ZIP Code, and census tract; however, the model specification should be tailored specifically to the outcome of interest.

## Data Availability

The datasets used and/or analyzed during the current study are available from CDC on reasonable request.

## Abbreviations

CDC: Centers for Disease Control and Prevention
CI: Confidence interval
DRH: Division of Reproductive Health
MRP: Multilevel regression with post-stratification
PhillyPRAMS: Philadelphia Pregnancy Risk Assessment Monitoring System
PRAMS: Pregnancy Risk Assessment Monitoring System
SAE: Small area estimation
